# CNN models discriminating between pulmonary micro-nodules and non-nodules from CT images

**DOI:** 10.1186/s12938-018-0529-x

**Published:** 2018-07-16

**Authors:** Patrice Monkam, Shouliang Qi, Mingjie Xu, Fangfang Han, Xinzhuo Zhao, Wei Qian

**Affiliations:** 1 Sino-Dutch Biomedical and Information Engineering School, Northeastern University, No. 195 Chuangxin Avenue, Hunnan District, Shenyang, 110169 China; 2 Key Laboratory of Medical Image Computing of Northeastern University (Ministry of Education), Shenyang, China; 3 College of Engineering, University of Texas at El Paso, 500W University, El Paso, TX 79902 USA

**Keywords:** Lung cancer management, Convolutional neural networks, Computed tomography (CT) images, Micro-nodules, Image classification

## Abstract

**Background:**

Early and automatic detection of pulmonary nodules from CT lung screening is the prerequisite for precise management of lung cancer. However, a large number of false positives appear in order to increase the sensitivity, especially for detecting micro-nodules (diameter < 3 mm), which increases the radiologists’ workload and causes unnecessary anxiety for the patients. To decrease the false positive rate, we propose to use CNN models to discriminate between pulmonary micro-nodules and non-nodules from CT image patches.

**Methods:**

A total of 13,179 micro-nodules and 21,315 non-nodules marked by radiologists are extracted with three different patch sizes (16 × 16, 32 × 32 and 64 × 64) from LIDC/IDRI database and used in the experiments. Three CNN models with different depths (1, 2 or 4 convolutional layers) are designed; their performances are evaluated by the fivefold cross-validation in term of the accuracy, area under the curve (AUC), F-score and sensitivity. The network parameters are also optimized.

**Results:**

It is found that the performance of the CNN models is greatly dependent on the patches size and the number of convolutional layers. The CNN model with two convolutional layers presented the best performance in case of 32 × 32 patches size, achieving an accuracy of 88.28%, an AUC of 0.87, a F-score of 83.45% and a sensitivity of 83.82%.

**Conclusions:**

The CNN models with appropriate depth and size of image patches can effectively discriminate between pulmonary micro-nodules and non-nodules, and reduce the false positives and help manage lung cancer precisely.

## Background

In 2017 in the United States, an estimated 222,500 people have been diagnosed with lung cancer, accounting for 13.2% of all new cancer cases [1]. In addition, the 5-year survival rate for lung cancer is less than 20%, and the 1-year survival rate is less than 50% [2]. This survival rate is strongly dependent on the development of the lung cancer before its detection. The earlier the lung cancer is diagnosed from a patient, the longer he or she will be susceptible to live.

Low-dose CT screening for lung cancer in individuals at high risk is recommended as an effective way of early detection by many scientific societies, based on the finding that it could reduce lung cancer mortality by 20% [3]. One of the aims of screening is to detect pulmonary nodules regarded as crucial indicators of lung cancer from CT images. Pulmonary nodules can be defined as small masses of tissue in the lung of round or oval shape, well-marginated with a diameter less or equal to 30 mm. Based on their diameters, pulmonary nodules can be divided into three categories including micro-nodules (< 3 mm), small nodules (3–9 mm) and nodules (10–30 mm).

Many automated pulmonary nodule detection systems have been developed to provide with a second opinion and aid the radiologist who is required to find nodules from a huge number of CT images [4]. Generally, these automated systems include two steps: (1) the candidate screening; (2) the false positive reduction [5]. The coarse candidates are screened by setting the threshold to the intensity and morphological parameters [6, 7]. The threshold value is usually lenient for high sensitivity, and a large number of false positives are generated. Hence, the advanced classifiers are required to decrease the false positive rate.

Some hand-crafted features and machine learning based classifiers have been employed to build up numerous automated pulmonary nodule detection systems. Hara et al. developed a 2nd order autocorrelation features based system for small nodules (< 7 mm) detection, achieving an accuracy of 94% [8]. Aggarwal et al. suggested a system based on image processing and segmentation techniques [9]. Santos et al. incorporated the Gaussian mixture models, Shannon’s and Tsallis’s Q entropy and support vector machine (SVM) into a system for detection and classification of small nodules (2–10 mm) [10]. Gong et al. eliminated the false-positive nodules utilizing the Fisher linear discriminant analysis (FLDA) classifier [11]. Liu et al. exploited the spatial fuzzy C-means (SFCM) and the random forest (RF) classifier [12]. Although the systems mentioned above achieved satisfactory performance, they comprise many steps and are computationally expensive.

Recently, the deep convolutional neural networks (CNNs) have been successfully used in many applications including the detection and classification of the pulmonary nodules. Deep CNN can automatically discover features from high-dimension data, which helps avoid the feature extraction and selection, and lead to the end-to-end solution [13]. Setio et al. proposed a multi-view CNN based system [7]. Tan et al. developed a two-phase framework system based on CNN for detection of juxta-pleural lung nodules and false positives reduction [14]. A 3D CNN based CAD system for detection of lung nodules in low dose CT images was proposed by Huang et al. [15]. Alakwaa et al. proposed a 3D-CNN based system for detection and classification of lung cancer, achieving an accuracy of 86.6% [16].

It is noted that previous studies are limited to small nodules with a diameter larger than 3 mm, no study on micro-nodules (diameter < 3 mm) has been done. It is reported that the pulmonary nodules with the diameter < 4 mm account for 59.5% in a total of 210 uncalcified pulmonary nodules [17]. Moreover, many recommendations have been given for the management of micro-nodules by different institutes. For example, the interval CT at 12 months is recommended for the subjects with high risk if the solid nodule (< 4 mm) is detected in the baseline scan, by Fleischner, Lung-RADS, and ACCP (American College of Chest Physicians) guideline [18].

In this paper, we propose to develop CNN models to discriminate between micro-nodules and non-nodules. As a pioneer work, we expand the automated pulmonary nodules detection to the micro-nodules. Due to the smaller size (diameter < 3 mm), the classification is thought to be different and more difficult than the large nodules. Our contributions or novelties are summarized as follows. First, without the nodule segmentation, hand-craft feature extraction and selection, the proposed CNN models provide with the end-to-end solution, i.e., from the image patches to the determination of micro-nodules or non-nodules. Second, a total of 13,179 micro-nodules and 21,315 non-nodules are extracted with three different patch sizes (16 × 16, 32 × 32 and 64 × 64) from LIDC/IDRI database, for both the validation of our CNN models and open access to future study. Third, the effect of the parameters optimization, the patches size (Receptive field), and the network depth on the performance of CNN models identifying the micro-nodules are clarified.

## Methods

### LIDC/IDRI dataset

All the CT images are acquired from a publicly accessible medical images database named the Lung Image Database Consortium and Image Database Resource Initiative (LIDC/IDRI) [19]. This database is made up of 1018 CT scans produced from 1010 different patients where the scans of eight patients had been duplicated by inadvertence. The obtained images are of sizes 512 × 512 and are kept in DICOM format.

Each scan was analyzed independently by four medical experts of different institutions with the unique aim of annotating the existing lung nodules. In the aim of achieving more accurate and efficient results, the annotation process was conducted in two steps. In the first step, every patient’s images file was examined and annotated by each of the four medical experts independently which is also called the “blinded reading”. In the second step, the results of the first step were put together and forwarded to each of the four medical experts which allowed them to see both their own annotations and the ones done by the other three colleagues. Making use of the information revealed by other medical experts’ markings, each medical expert for the second time examined and annotated each scan independently and made a final decision about the existing lung nodules. This second step of the annotation process is also known as “unblinded reading”.

**Fig. 1:**
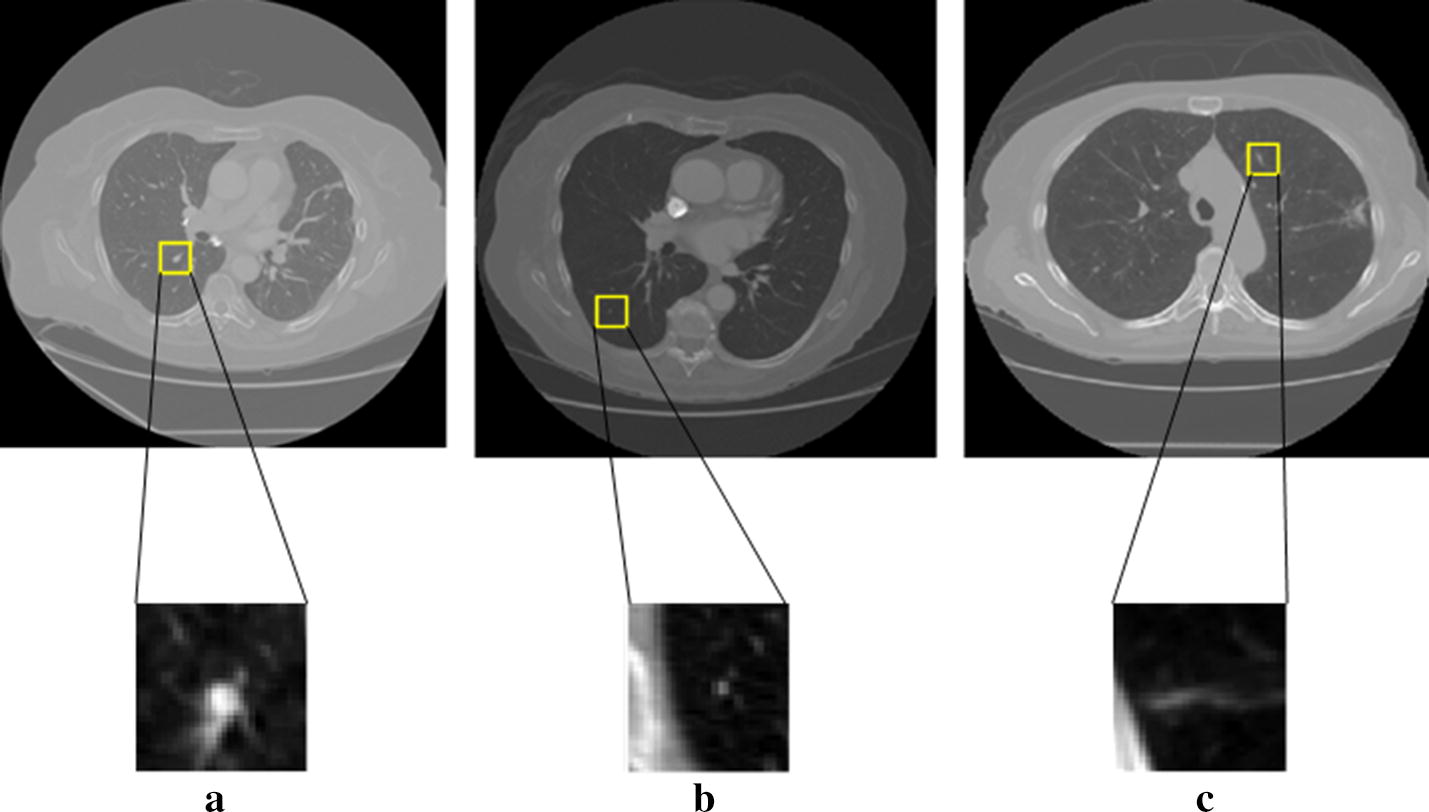
Examples of the suspected lesions and non-nodules identified in the LIDC/IDRI dataset. **a** Nodules (3 mm ≤ diameter < 30 mm); **b** micro-nodules (diameter < 3 mm); **c** non-nodules (3 mm ≤ diameter)

According to their diameters, the medical experts classified the found lesions into three main categories: (1) nodules (3–30 mm). This type of nodules is having their outline well marked by each of the medical expert. An example of lung CT image containing a nodule is shown in Fig. 1a. (2) Micro-nodules (< 3 mm). There is no provided contour for this type of nodules. They are pointed out only through their three-dimensional center-of-mass. Figure 1b illustrates a micro-nodule found in a lung CT image. (3) Non-nodules (> or equal 3 mm). They are also pointed out only through their three-dimensional center-of-mass. The presence of non-nodule in a lung CT image is demonstrated in Fig. 1c.

### Image patches with different sizes

To avoid the data duplication, the eight duplicated patients’ scans of the LIDC/IDRI dataset were ignored. Every lesion detected by the medical experts is represented in the xml file by a lesion identifier, a ROI (region of interest) including the x, y and z coordinates. Both the micro-nodules and the non-nodules are represented by only one point with no reported characteristics. There is a unique keyword “Locus”, proper to non-nodules in replacement of “edge map” in the definition of nodules and micro-nodules.

**Fig. 2:**
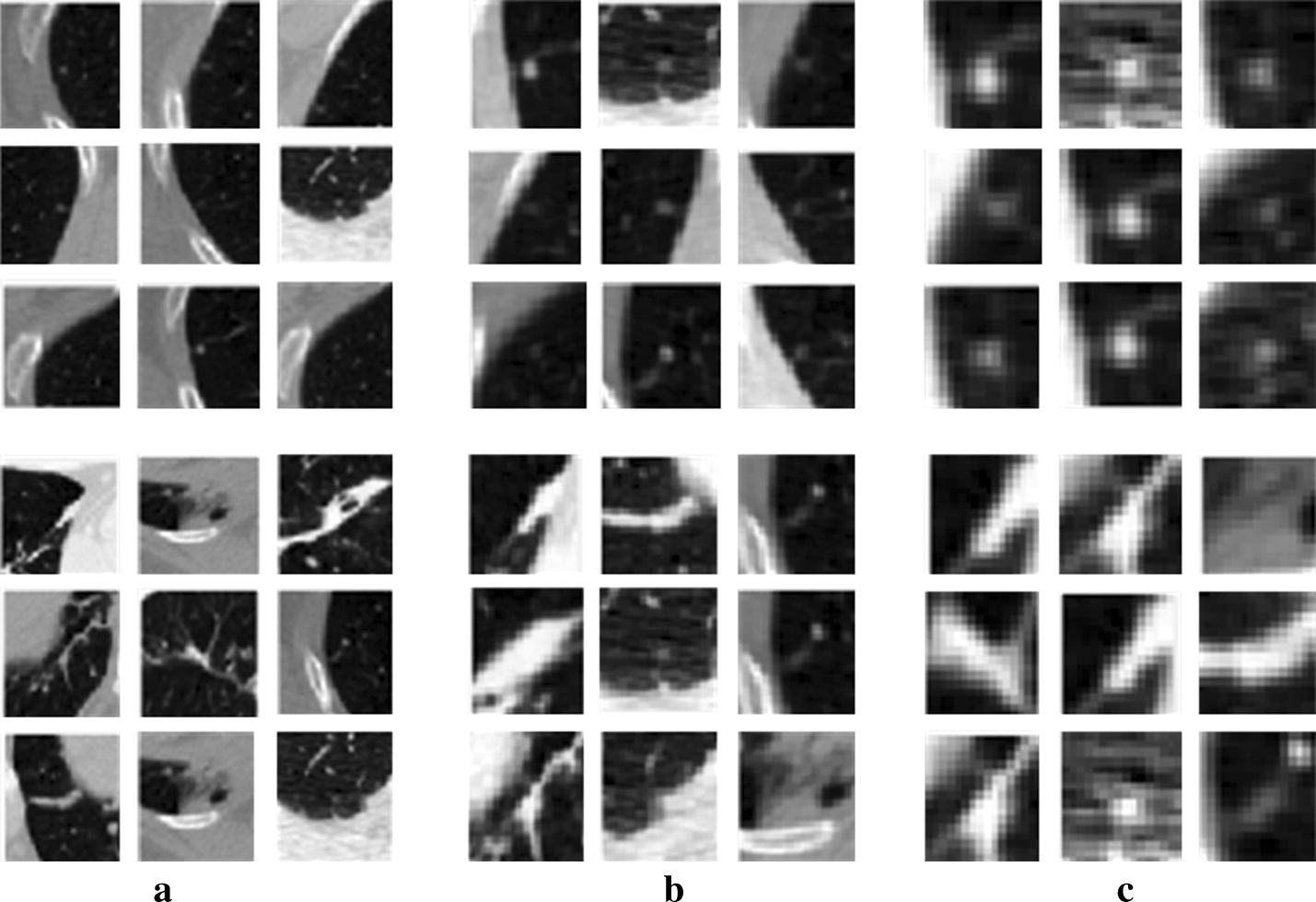
The extracted patches of micro-nodules (the first row) and non-nodules (the second row) with different sizes. **a** With the patch size of 64 × 64; **b** with the patch size of 32 × 32; **c** with the patch size of 16 × 16

For our CNN models, the input is the small patches centering on the point defined by xml file and cropped from the CT images. The patch size, also named the receptive filed of a network, determines the surrounding range of micro-nodules and non-nodules. Because of the small size of micro-nodules, the discriminating features learned in the CNN models are strongly dependent on the surrounding or contextual information of the micro-nodules. Thus, to determine the appropriate patch size, we generated image patches with different sizes including 16 × 16, 32 × 32 and 64 × 64. Some examples of the micro-nodules and non-nodules are presented in Fig. 2, in the form of different patch sizes. Finally, a total of 13,179 micro-nodules and 21,315 non-nodules image patches were extracted.

### CNN models

**Fig. 3:**
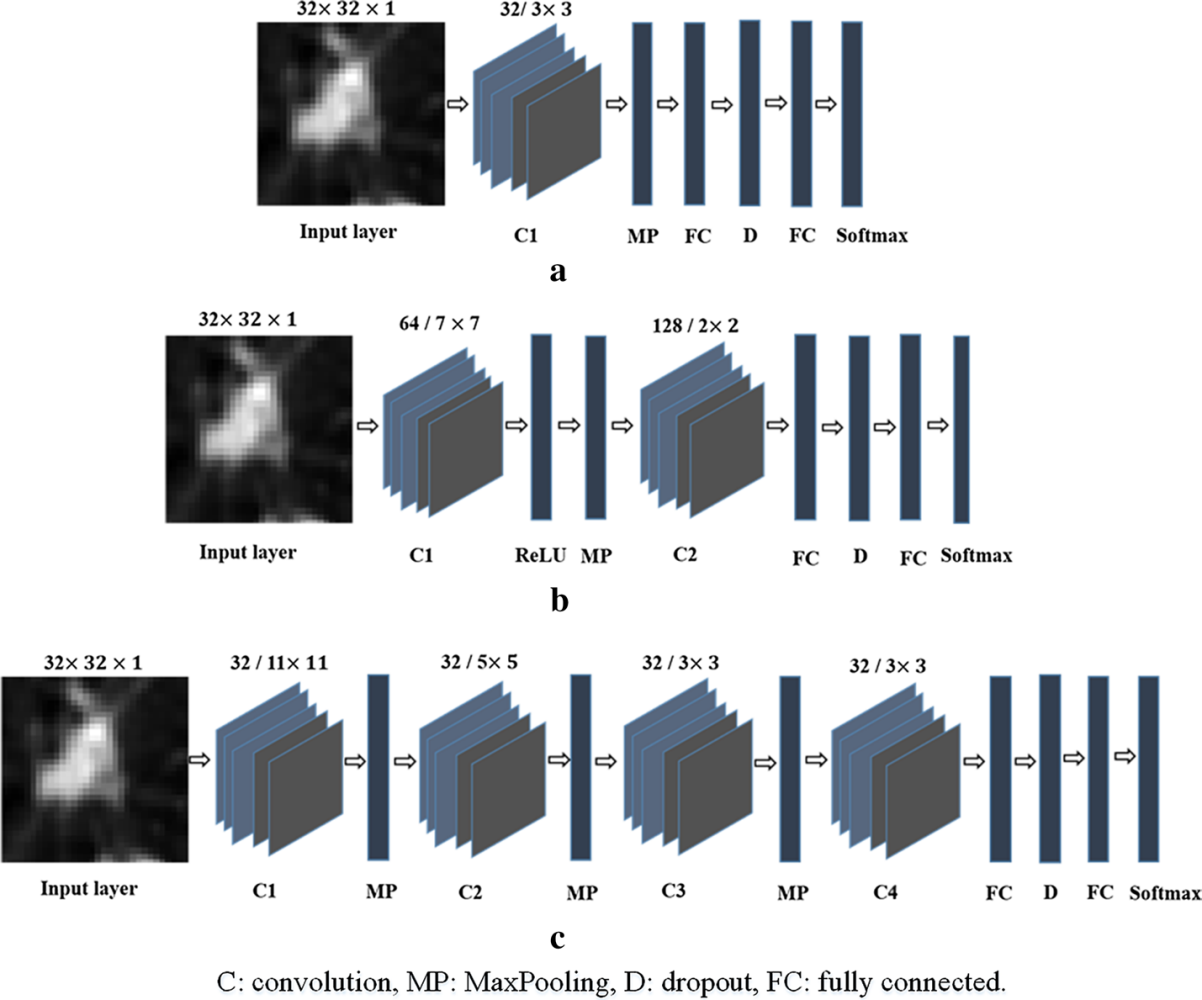
The architectures of the proposed three CNN models. **a** The first CNN model (M1); **b** the second CNN model (M2); **c** the third CNN model (M3)

Considering the fact that the micro-nodules and non-nodules are both tiny objects, we designed three CNN models with small filters and different depths, as shown in Fig. 3. The first CNN model (M1) consists of one convolutional layer and one MaxPooling layer after which follow a fully connected layer, a dropout layer and two fully connected layers including the Softmax layer. The dropout layer helps the network in ignoring some units during the training process which can overcome the problem of overfitting.

The second CNN model (M2) consists of two convolutional layers. The first one is followed by a rectified linear activation (ReLU) layer and a MaxPooling layer. A dropout layer and two fully connected layers including the Softmax layer follow the second convolutional layer. The ReLU layer plays the role of accelerating the convergence of the stochastic gradient descent (SGD) resulting to an improvement of the training speed.

The architecture of the third CNN model (M3) comprises four convolutional layers in which the first, second and third ones are followed by a MaxPooling layer which helps the network to focus only on the image information resulted from the convolution process [21]. The last convolutional layer is followed by a dropout layer and two fully connected layers including the Softmax layer.

### CNN training and cross-validation experiments

The fivefold cross-validation experiments were carried out for each of the three proposed CNN models and each of the three patch sizes. That is, after dividing all data into 5 disjoint subsets, 4 subsets are added to the training set, and the remaining is used for testing. For the training process, we set the learning rate of 0.0001, the parameters of momentum of 0.9 and the epochs’ number of 50. The number of epochs and the kernels size are varied to optimize the network parameters.

All the experiments were implemented in Matlab 2018a under a Windows 10 operating system on a workstation with CPU Intel Xenon E5-2640 v4 @ 2.40 GHz, GPU NVIDIA Quadro M4000 and 32G RAM.

### Evaluation metrics

To evaluate the performance of the proposed CNNs, we used four metrics such as the average F-score also known as F-score, the accuracy, the sensitivity or recall (true positive rate) and the area under the curve (AUC). The average F-score is a measure computed from considering both the precision and the recall of the data samples and is expressed as follows:

1 $$ F-score = \sum\limits_{C = 1}^{2} {\frac{{Recall_{C} \times Precision_{C} }}{{Recall_{C} + Precision_{C} }}} $$

where the number of classes *C* is equal to two. The precision and recall are defined as

2 $$ Precision = \frac{TP}{TP + FP} $$

3 $$ \text{Recall} = \frac{TP}{TP + FN} $$

where *TP* (true positive) is the proportion of the samples correctly classified, *FP* (false positive) is the proportion of samples shown correctly classified as belonging to a specific class when they actually do not belong to that class, and *FN* (false negative) represents the number of samples classified as not belonging to a specific class when they actually do belong to that class.

In the proposed classification method, making use of the labels previously assigned to each micro-nodule and non-nodule image patch (1—micro-nodule; 0—non-nodule), the TP rate and the true negative (TN) rate are obtained by determining the number of micro-nodules correctly labeled with one and the number of non-nodules correctly labeled with zero respectively. Similarly, the FP rate and the FN rate consist of the number of non-nodules labeled with one and the number of micro-nodules labeled with zero respectively.

The accuracy can be defined as an evaluation measure computed by dividing the amount of data correctly classified by the overall data used for the experiments. The AUC is equal to the probability that a classifier will rank a randomly chosen positive instance higher than a randomly chosen negative example. It is computed from the receiver operating characteristic (ROC) curves.

## Results

### Networks parameters optimization

For the first CNN model (M1) and the image patches of 32 × 32, the kernel size of the convolutional layer was chosen to be 3 × 3, yielding the highest values of 83.8%, 0.84, 78.54%, and 81.26% for the accuracy, the AUC, the F-score, and the sensitivity, respectively. Actually, we started the training with the kernels of size 11 × 11 and noticed that smaller sizes of the kernel led to an improvement of the accuracy, AUC, F-score and sensitivity.

**Fig. 4:**
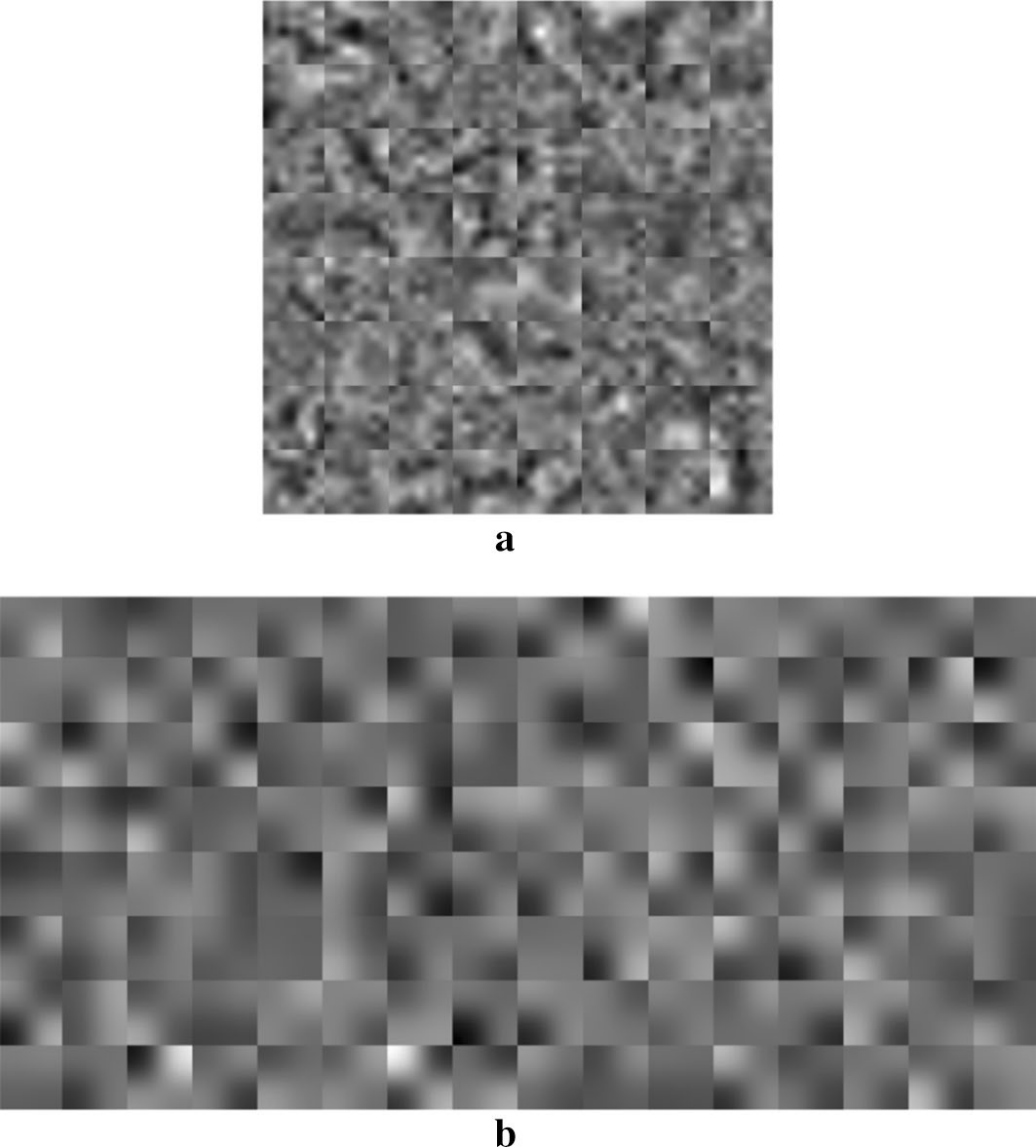
The visualization of the learned features in the trained second CNN model (M2). **a** The smoothed 64 kernels (7 × 7) in the first convolutional layer; **b** the smoothed 128 kernels (2 × 2) in the second convolutional layer

Similarly, the second CNN model (M2) achieved an accuracy of 88.28%, an AUC of 0.87, an F-score of 83.45% and a sensitivity of 83.82%. In the beginning, we considered 50 for the number of epochs; then we noticed that its increase resulted in an improvement of both the accuracy and the F-score. Therefore, we chose the optimal epochs’ number to be 120 whose increase did not improve the training performance. The micro-nodules being of very small size, we shrunk the kernels of the second convolutional layer to the size of 2 × 2; which resulted in an accurate capture of the fine image details leading to the improvement of the training results. In addition, Fig. 4a, b display the visualization of the learned kernels in the first (7 × 7) and second (2 × 2) convolutional layers, respectively. It can be seen that there is no presence of noises and artifacts. The image patterns are very smooth and contain much more feature information; which demonstrates the good choice of the network parameters resulting to a well-trained network without overfitting and yielding great classification performance.

The values adopted for the parameters of the third CNN model (M3) were 0.0001, 0.9 and 50 for the learning rate, the momentum parameters and the epochs’ number, respectively; yielding an accuracy of 86.84%, an AUC of 0.86, an F-score of 82.79%, and a sensitivity of 83. 67%. We noticed no improvement of the training results while assigning different values to those parameters.

### CNN models with different patch sizes

**Fig. 5:**
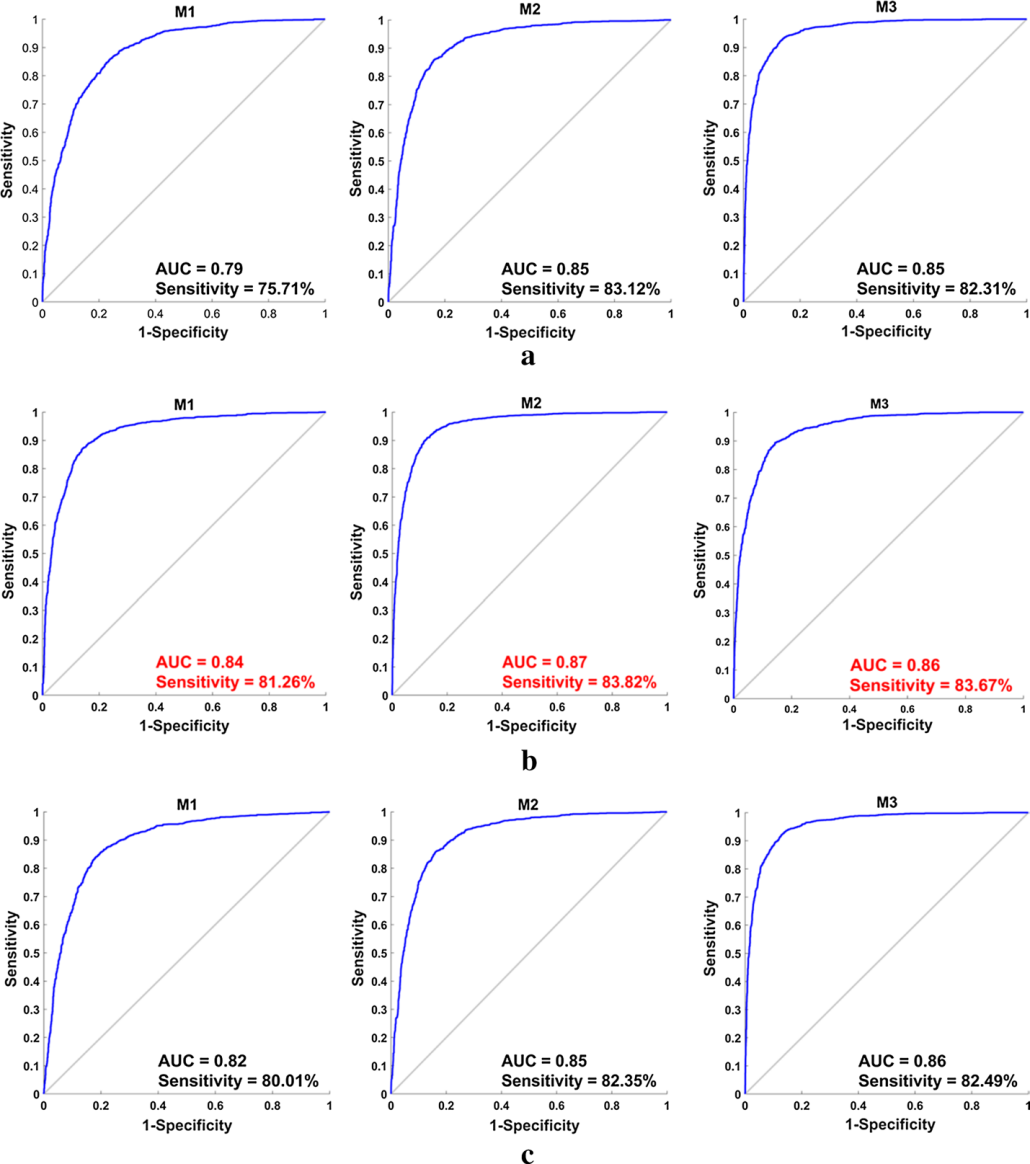
The performance evaluation of the proposed CNN models with the input image patches of different sizes. **a** The patch size of 64 × 64; **b** the patch size of 32 × 32; **c** the patch size of 16 × 16

For three patch sizes of 64 × 64, 32 × 32, 16 × 16, the first CNN model (M1), the second CNN model (M2), and the third CNN model (M3) achieved different F-score, accuracy, sensitivity and AUC, as shown in Table 1. In addition, the ROC curves are drawn in Fig. 5 with the sensitivity and the AUC. From left to right, the first, second and third column correspond to the classification performance of M1, M2 and M3 in term of AUC and sensitivity, respectively.

**Table 1:** Performance of three CNN models for three different patch sizes

	Patch sizes	F-score (%)	Accuracy (%)	Sensitivity (%)	AUC (%)
The first CNN model (M1)	64 × 64	75.09	81.22	75.71	79.99
	*32 × 32*	*78.54*	*83.8*	*81.26*	*84.66*
	16 × 16	81.09	85.65	80.01	82.74
The second CNN model (M2)	64 × 64	81.84	86.41	83.12	85.64
	*32 × 32*	*83.45*	*88.28*	*83.82*	*87.37*
	16 × 16	84.3	87.03	82.35	85.65
The third CNN model (M3)	64 × 64	81.92	86.35	82.31	85.36
	*32 × 32*	*82.79*	*86.84*	*83.67*	*86.73*
	16 × 16	83.53	87.504	82.49	86.17

The rows marked in italics illustrate the patch size achieving the highest sensitivity and AUC in the specific model

We started the experiments with patches of size 64 × 64 and we noticed an improvement of the performance of all the three CNN models while choosing smaller patches size (32 × 32), as described by the accuracy, the F-score, the sensitivity and the AUC values recorded in Table 1. A progressive decrease of the patches size till 16 × 16 led to an increase of the F-score and the accuracy values but resulted to a smaller value of the AUC and the sensitivity. Therefore, the appropriate patches size was chosen to be 32 × 32.

### CNN models with different depths

**Fig. 6:**
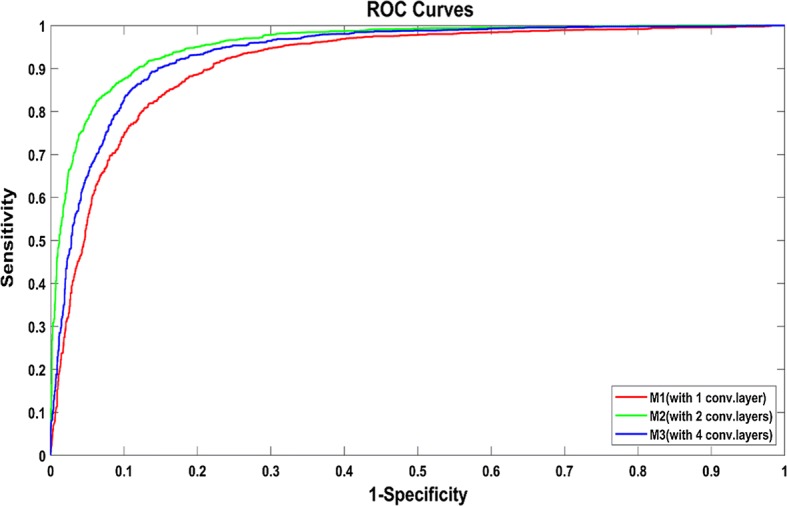
Receiver operating characteristic (ROC) curves of CNN models for micro-nodules and non-nodules classification with different depths (1, 2 or 4 convolutional layers) (the size of the input image patches is 32 × 32)

For the patch size of 32 × 32, we performed experiments with three different depths and their performances are presented in Table 1 and Fig. 6. One can find that M2 being deeper than M1 performed better, as illustrated by their respective accuracy, AUC, F-score and sensitivity values. Moreover, by comparing the closeness of the ROC curve peak to the value one and the corresponding AUC values, M2 outperforms M3 even though M3 architecture includes more layers. Therefore, the second CNN model (M2) is the most suitable CNN architecture for discriminating between micro-nodules and non-nodules.

**Fig. 7:**
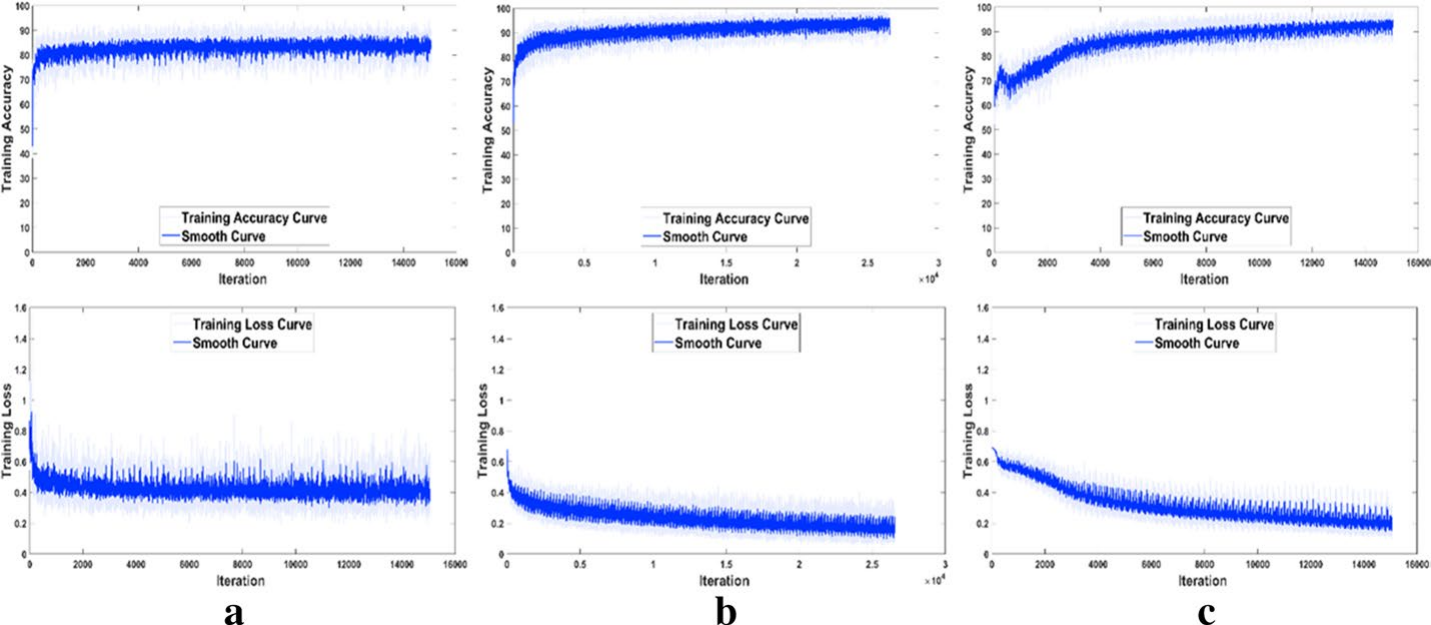
The training accuracy and loss functions of the three proposed CNN models. **a** M1 (with 1 convolutional layer); **b** M2 (with 2 convolutional layers); **c** M3 (with 4 convolutional layers)

The training accuracy and loss functions of M1, M2 and M3 are plotted in Fig. 7, where smoothing was performed for better analysis of the curves. It can be found that M1 converges very fast, but the accuracy can hardly reach 90% and the loss value remains high (> 0.30). M2 needs more iterations, and the accuracy is the highest (almost 100%) and the loss value is the lowest (0.56) among the three models. For M3, the convergence is not stable, and the loss value can only decrease to 0.76.

### Classification results and comparison with previous works

**Fig. 8:**
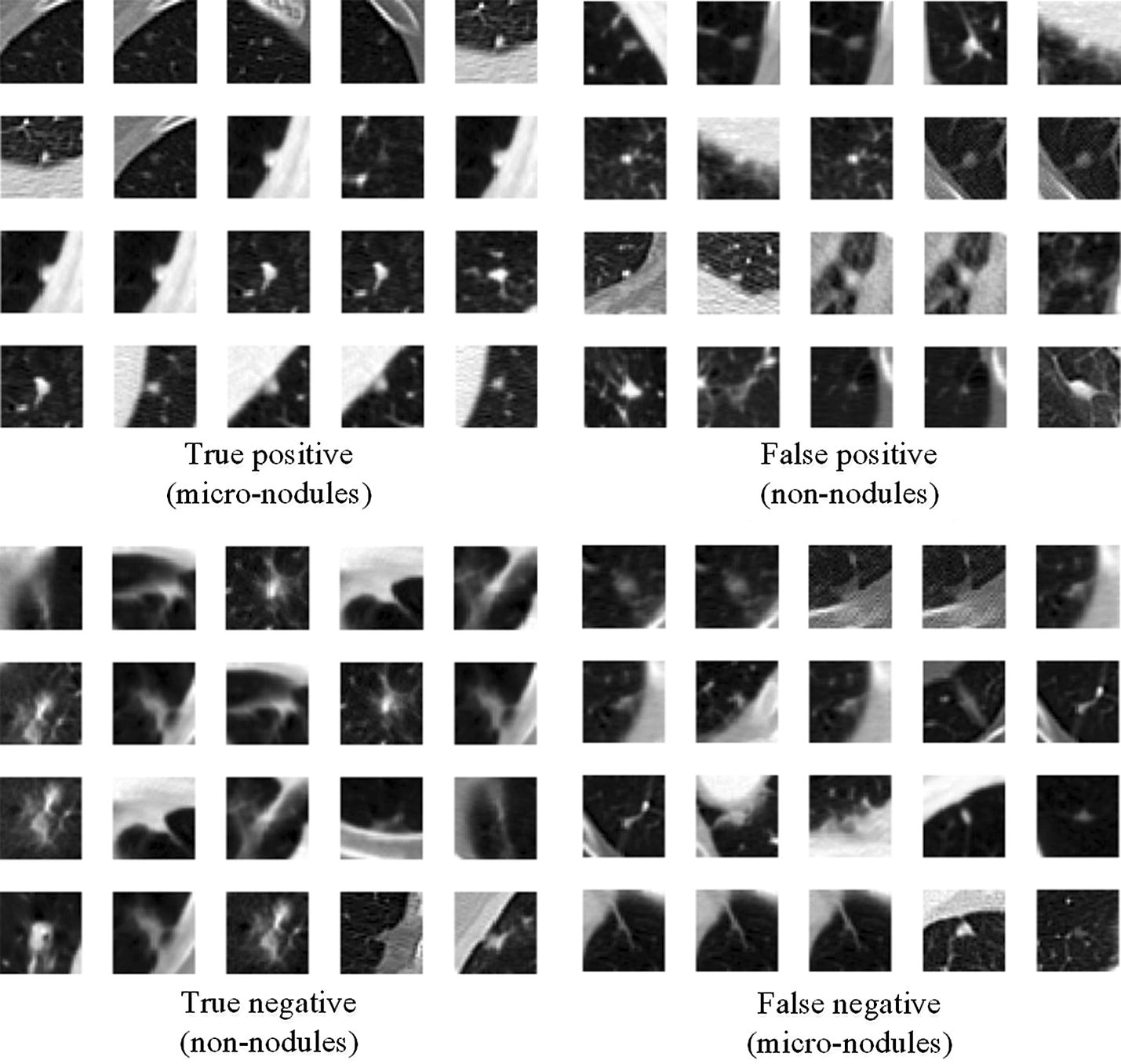
Examples of the image patches classified by the proposed CNN model (M2) with the input patches of 32 × 32

Some image patches after the classification using M2 are shown in Fig. 8. As can be observed, micro-nodules and non-nodules of our dataset are very similar in both sizes and shapes whose features can sometime be very difficult to differentiate; which might easily lead to misclassification. However, the second CNN model (M2) is robust and efficient enough to achieve the best classification performance with relatively low false positive rate justified by the highest sensitivity of 83.82%.

We have drawn a comparison with some existing works done on the LIDC/IDRI dataset, as shown in Table 2. We have analyzed all the 1010 patient images files of the LIDC/IDRI database without excluding any cases which is significantly higher than the number of scans considered by most of the existing works. On the other hand, we have extracted a total of 13,179 micro-nodules where more than 2600 were used for testing which shows the high complexity of detection due to a more diversified dataset. Although Golan et al. [21] have used a total of 1018 scans, their test set comprises only 204 samples yielding a sensitivity even smaller than that of our system. Jiang et al. conducted their study on 1006 scans achieving a sensitivity of 80.06% with 4.7 false positives per scan and 94% with 15 false positives per scan [22]. Therefore, our system outperforms the Golan et al. [21] and Jiang et al. [22] systems in both number of study cases and achieved sensitivity. Furthermore, other systems only focused on nodules with diameter ≥ 3 mm while our system aimed to classify much smaller nodules (diameter < 3 mm) making it more difficult.

**Table 2:** The performance comparison between the proposed model and some existing models

Models	Year	Number of scans	The nodule size	The number of nodules	Sensitivity
Our model	–	1010	Diameter < 3 mm	2635	83.82%
Jiang et al. [20]	2017	1006	Diameter > 3 mm	–	80.06% (with 4.7 false positives per scan) 94.00% (with 15.1 false positives per scan)
Golan et al. [12]	2016	1018	Diameter ≥ 3 mm	204	78.9% (with 20 false positives per scan) 71.2% (with 10 false positives per scan)

## Discussion

In this study, we have developed three CNN models to differentiate micro-nodules and non-nodules from CT images. These models can be used to reduce the false positives in automated pulmonary nodule detection, which will consequently reduce the radiologists’ workload, avoid unnecessary anxiety for the affected subjects, and help imply more accurate follow-up leading to proper treatment and lives saving.

### The significance of the accurate recognition of micro-nodules

The survival outcome of an individual suffering from lung cancer is strongly dependent on the stage of the cancer when it is diagnosed; which is mostly evaluated based on the size of the pulmonary nodules shown on CT scans. Recently, numerous works have been conducted aiming to accurately evaluate the pulmonary nodules, which is of significant importance in the therapeutic decision [23, 24]. However, it is still very challenging to recognize nodules of diameter < 3 mm.

For the identified micro-nodules, many specialized lung cancer associations had given various recommendations of their management such as “to be ignored” or “to be kept under surveillance”. For populations in Asia, annual CT scans are also mentioned depending on clinical judgment and patient preference for subjects with solid nodule less than 4.0 mm diameter [23]. For the multiple well-defined ground-glass nodules (GGN) with the diameter of 5 mm or less, the conservative management of follow-up CT scans at 2 and 4 years are recommended [24]. Moreover, there is no consensus about the malignancy risk of micro-nodules. MacMahon et al. reported that the malignancy risk is highly related to its size: 0.2% for nodule < 3 mm, 0.9% for nodules 4–7 mm, and 18% for 8–20 mm [25]. However, Munden et al. found that 28% of small pulmonary nodules detected at baseline CT scan will increase in size, indicating metastatic disease [26].

### CNN models or the hand-crafted features based classifier or their fusion

In general, there are three strategies to discriminate between pulmonary micro-nodules (or small nodules) and non-nodules from CT images. First, the CNN models are used to avoid some computationally expensive steps such as the segmentation, feature extraction and selection, and lead to the end-to-end solution. Similarly, the residual CNN has been employed to reduce the false positives [27], the massive-training artificial neural networks (MTANNs) have also been used to build up the end-to-end machine-learning models given the limited training data [28]. To the end of addressing the lack of data problem, the data augmentation and transfer learning have been proposed [29]. We had tried to do data augmentation (i.e., rotation, translation, and scale) in this study, but no significant improvement was found for any performance measures (i.e. the F-score, accuracy, sensitivity, and AUC). Since we had a total of 13,179 micro-nodules and 21,315 non-nodules image patches, the transfer learning was not considered. Other end-to-end models such as the deep belief network (DBN) and stacked denoising autoencoder (SDAE) might also be included into this category [30].

Second, the hand-crafted features based classifiers have been widely applied. Based on boundary delineation or segmentation, various features have been considered including intensity, morphology, texture, wavelets, and so on [31–33]. Then the feature selection and machine learning based methods are followed [34, 35]. For example, to characterize the lung cancer phenotypes, Parmar et al. [36] had evaluated 14 feature selection algorithms and 12 classification methods, and found that Wilcoxon test based feature selection method and random forest achieved the highest performance.

Third, the fusion of CNN estimation and hand-crafted features has also been used to address the false-positive reduction in the automated detection of nodules [4]. Although it is difficult to decide on the best strategy to be applied, our CNN models present one end-to-end solution with satisfied performance for the classification of micro-nodules and non-nodules.

### CNN parameters optimization

The choice of the network architecture and of the training parameters may have great effect on the classification results. It has been suggested that a wise network parameters (number of magnitude M) selection allows designing a network producing accurate results [37]. For instance, He et al. proposed ResNet with 152 layers and only 2 M parameters [38]; which significantly outperformed VGG-Net [39] consisting of 16 layers and 140 M parameters. Regarding the kernel sizes of the convolutional layers, we have noticed that small kernels (3 × 3 or 2 × 2) can access the fine details of the images leading to a more accurate features discovering. In 2014, one of the top networks of the ILSVRC was the VGG-Net with kernels of 3 × 3 developed by Simonyan et al. [39]. In our models, a decrease of the kernel sizes led to an improvement of the system performance where 2 × 2 kernels yielded very satisfactory classification results.

While designing a CNN network, it is necessary to include a dropout layer and an activation layer because they can be used to overcome the problems of overfitting and high computation time. Besides, the learning rate and the epoch number are critical parameters whose values must be assigned carefully and in regard with the problem at hands.

### Patch size for small objects

An input of large images may take a longer time and result in a poor features learning especially for the case of tiny objects. The computation capacity of the numerous existing deep learning based methods is conditioned by the memory available on the Graphics Processing Units (GPUs), making it almost impossible to apply deep CNN based methods for processing very large images [37]. Therefore, to the end of remedying to the memory requirement, it is necessary to split the large images into small patches comprising the objects whose features need to be detected. Additionally, large image patches may contain important amount of unnecessary information which could cause mixed-pixel problem [40].

In our system, we have investigated the image patches of size 16 × 16, 32 × 32 and 64 × 64. Given the small size of the micro-nodules, the image patches of 32 × 32 do not contain too much unnecessary information; which helps the CNN to accurately extract the features indispensable for recognizing the micro-nodules. Thus, the appropriate patch size was chosen to be 32 × 32.

### Is the larger depth of the CNN always good?

Recently, the number of layers of deep CNNs has become larger and larger. The first successful application of CNN in recognition task was achieved with AlexNet proposed by Krizhevsky et al. [41]. This network architecture consisted of eight layers. Later on, some much deeper CNN structures were proposed, including GoogLeNet [42], VGG-Net [39] and the Residual Network (ResNet) and its variants [38, 43]. It is beneficial to increase the number of layers in the network because the features can be easily learned at different abstraction levels. However, using very deep network structures requires more parameters to be learned leading to an increase of the network complexity, the training time, the error generalization and the overfitting rate.

In our study, we have explored CNNs with one, two, and four convolutional layers; where the most successful model was not the deepest one but the one with two convolutional layers. Therefore, the deeper CNN structures are not always good and it is crucial to take into consideration the following factors while designing a network: the size and shape of the objects to be classified, the available dataset, and some parameters such as kernels size and not only rely on the network depth. The study conducted by Tajbakhsh et al. [28] supported this point, and in our previous work, we developed an agile CNN model with effective depth through combining the LeNet and AlexNet [44].

### Limitations and future works

Although our proposed CNN models with appropriate depth and size of image patches can effectively discriminate between pulmonary micro-nodules and non-nodules, there are still some limitations to be addressed. First, the CNN models are based on the 2D image patches. Actually, one can find that it is really a difficult task to discriminate between some micro-nodules and non-nodules using the 2D visual characteristics, as shown in Fig. 8. The 3D CNN models or RNN models considering more contextual information will be explored in the next work. Second, we did not combine the patches (or the receptive field) with different size together as done by Dou et al. [5]. Third, we only used the dataset of LIDC, the generalizability of our CNN models is not known for other independent dataset. These limitations will be addressed in the future study.

## Conclusion

The proposed CNN models with appropriate depth and size of image patches can effectively and efficiently discriminate between pulmonary micro-nodules (diameter < 3 mm) and non-nodules, and decrease the false positive rate. For the tiny objects, small image patches (or the receptive field) might lead to high performance. The deeper CNN structures are not always good and it is crucial to consider the dataset and the objects of interest while finding the effective depth. Some parameters such as kernels size and the number of epochs require to be optimized. These methodological findings and the extracted dataset of micro-nodules and non-nodules might help design other CNN models. The proposed CNN models might help reduce the radiologists’ workload and unnecessary anxiety for the affected subjects; and contribute to a precise management of lung cancer through early and automatic detection of pulmonary nodules from CT images.
